# Factors Differentiating the Antioxidant Activity of Macular Xanthophylls in the Human Eye Retina

**DOI:** 10.3390/antiox10040601

**Published:** 2021-04-14

**Authors:** Justyna Widomska, Wieslaw I. Gruszecki, Witold K. Subczynski

**Affiliations:** 1 Department of Biophysics, Medical University of Lublin, Jaczewskiego 4, 20-090 Lublin, Poland; 2 Department of Biophysics, Institute of Physics, Maria Curie-Sklodowska University, 20-031 Lublin, Poland; wieslaw.gruszecki@umcs.pl; 3 Department of Biophysics, Medical College of Wisconsin, 8701 Watertown Plank Road, Milwaukee, WI 53226, USA; subczyn@mcw.edu

**Keywords:** zeaxanthin, lutein, macular xanthophyll, lipid bilayer, fovea

## Abstract

Macular xanthophylls, which are absorbed from the human diet, accumulate in high concentrations in the human retina, where they efficiently protect against oxidative stress that may lead to retinal damage. In addition, macular xanthophylls are uniquely spatially distributed in the retina. The zeaxanthin concentration (including the lutein metabolite meso-zeaxanthin) is ~9-fold greater than lutein concentration in the central fovea. These numbers do not correlate at all with the dietary intake of xanthophylls, for which there is a dietary zeaxanthin-to-lutein molar ratio of 1:12 to 1:5. The unique spatial distributions of macular xanthophylls—lutein, zeaxanthin, and meso-zeaxanthin—in the retina, which developed during evolution, maximize the protection of the retina provided by these xanthophylls. We will correlate the differences in the spatial distributions of macular xanthophylls with their different antioxidant activities in the retina. Can the major protective function of macular xanthophylls in the retina, namely antioxidant actions, explain their evolutionarily determined, unique spatial distributions? In this review, we will address this question.

## 1. Introduction

It is accepted that the yellow pigmentation of the macula lutea is a consequence of the selective accumulation of two dietary xanthophylls, namely zeaxanthin (3R,3′R-zeaxanthin) and lutein, in the human retina [1]. The third macular xanthophyll present in the retina, meso-zeaxanthin (3R,3′S-zeaxanthin), is formed by the enzymatic transformation of lutein molecules. Meso-zeaxanthin is not found in significant amounts in the typical diet compared to zeaxanthin and lutein and is undetectable in the blood serum. However, it has been found in trace amounts in fish skin, shrimp shells, and turtle fat [2,3,4,5]. It should be noted here that the structure and properties of meso-zeaxanthin are closer to those of zeaxanthin than to those of lutein; however, meso-zeaxanthin is a different molecule to zeaxanthin. All these nutrients (meso-zeaxanthin, zeaxanthin, and lutein) are used as supplementary interventions for individuals with low-xanthophyll diets and who are at risk for age-related macular degeneration (AMD) [6,7,8,9,10,11]. Additionally, they have been found to positively correlate with a range of cognitive functions in older adults [12,13,14].

The retinal spatial distributions are different for zeaxanthin, meso-zeaxanthin, and lutein, and the meso-zeaxanthin and zeaxanthin-to-lutein ratio changes as the eccentricity moves away from the fovea [15,16,17]. The gold-standard method for quantitative analysis of the zeaxanthin/lutein molar ratio is the high-performance liquid chromatography (HPLC) technique. The first HPLC analysis of human retina extracts showed that the zeaxanthin-to-lutein ratio at the foveal center is approximately 2.4:1 [15]. In later HPLC studies, Bone and coauthors could separate two forms of stereoisomers of zeaxanthin, and their research showed that the meso-zeaxanthin-to-zeaxanthin-to-lutein ratio is 1:1:1 at the fovea center [16]. Recently, confocal Raman spectroscopy was applied to image the human retina and map the different spatial distributions of zeaxanthin and lutein [17]. Because carotenoids give a relatively strong Raman scattering signal, generated from the oscillation of double bonds in their conjugated isoprenoid chain, and because zeaxanthin and lutein differ in the length of their conjugated double bonds, they have subtly distinguishable resonance Raman spectra [17,18,19]. Confocal resonance Raman microscopy can distinguish lutein from zeaxanthin, but not zeaxanthin from meso-zeaxanthin. The resonance Raman spectra of the macular xanthophylls collected during retinal imaging confirmed that zeaxanthin is selectively concentrated in the fovea and its concentration decreases with increasing eccentricity from the fovea. In contrast, lutein is distributed more randomly. Li et al. reported a higher meso-zeaxanthin and zeaxanthin-to-lutein ratio (4:1) [17] at the fovea than reported previously (2:1) with HPLC-based studies [15]. Both the HPLC and resonance Raman spectroscopy methods have their advantages and disadvantages in the identification of macular xanthophylls in human retina sections [20,21,22], but both showed the selective accumulation of zeaxanthin and meso-zeaxanthin in the center of the fovea. Not only did the resonance Raman spectroscopy imaging method separately map the spatial distributions for zeaxanthin and lutein, but it also measured the retinal layer distribution for both of these macular xanthophylls. Macular carotenoids were mainly concentrated in the outer and inner plexiform layers, which is consistent with a previous study by Snodderly et al. [23]. Two important questions about macular carotenoids remain unanswered: the first question concerns the reason for the non-uniform distribution of lutein and zeaxanthin in the macula lutea. The second question deals with the replacement of dietary lutein with meso-zeaxanthin. However, both questions emphasize the preferential accumulation of zeaxanthin (3R,3’R-zeaxanthin and 3R,3´S-zeaxanthin) in the most vulnerable region of the fovea. It is well accepted that these three macular xanthophylls (zeaxanthin, lutein, and meso-zeaxanthin) represent an important part of the antioxidant defense system of the human retina. A number of studies have identified four photoprotective mechanisms of xanthophyll action: (i) they scavenge reactive oxygen species (ROS), (ii) they suppress free radical chain reactions, (iii) they attenuate blue light due to absorption in the short-wavelength region, and (iv) they are able to quench the excited triplet state of photosensitizers. The preferential accumulation of zeaxanthin in the center of the fovea suggests that it has a higher antioxidant capacity as compared with lutein, because this region is exposed to intense light and has a high metabolic rate. It is known that vitamin E and zeaxanthin are similar in their spatial distributions, and both show maximum concentrations in the central part of the fovea [24]. Whether the synergy between zeaxanthin and other retinal antioxidation systems is more efficient than between lutein and other retinal antioxidants should be determined. In the future, an investigation should be done to determine whether zeaxanthin is a more potent lipid antioxidant than lutein. A comparison of the capacity to quench singlet oxygen by zeaxanthin and lutein in organic solutions has been presented in numerous studies [25,26,27,28]. However, the quenching of singlet oxygen by macular xanthophylls in a lipid environment has not been studied intensively [29,30,31]. A multitude of factors affect the ability of carotenoids to quench singlet oxygen in the lipid membrane in vitro and in vivo; for example, the different (i) tendency to form aggregates, (ii) tendency of the photosensitized oxidation of carotenoid molecules to result in the formation of endoperoxides and aldehydes, (iii) orientation in the lipid bilayer, (iv) distribution in the lipid bilayer, and (v) synergistical cooperation with other retinal antioxidants. Mapping zeaxanthin in the retina indicates that zeaxanthin—not lutein—is the more important carotenoid of the fovea. In this review, we address the following question: in the fovea, is zeaxanthin a better lipid antioxidant than lutein?

## 2. Physical Antioxidant Actions of Macular Xanthophylls

### 2.1. Absorption Spectra and Blue Light Filtration

**Figure 1:**
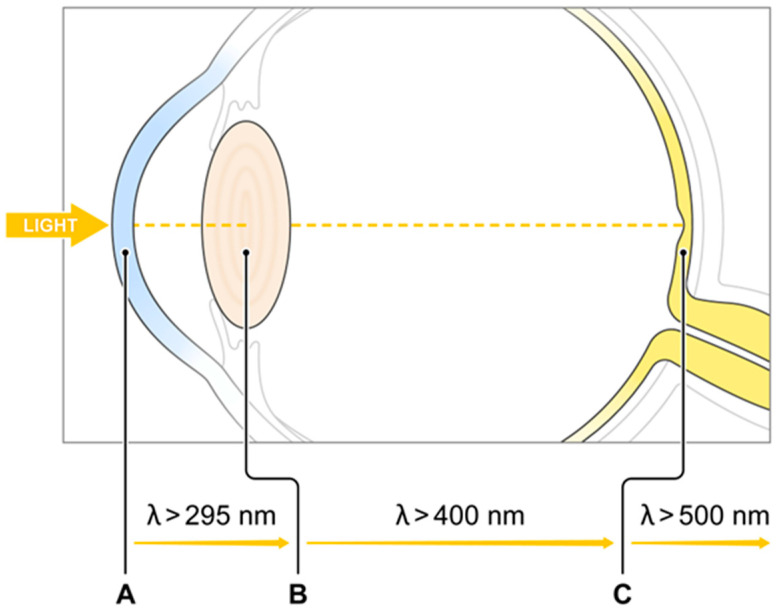
Schematic illustration of the attenuation of ultraviolet and blue light transmission by human ocular filters (A—cornea, B—lens, C—macular xanthophylls).

**Figure 2:**
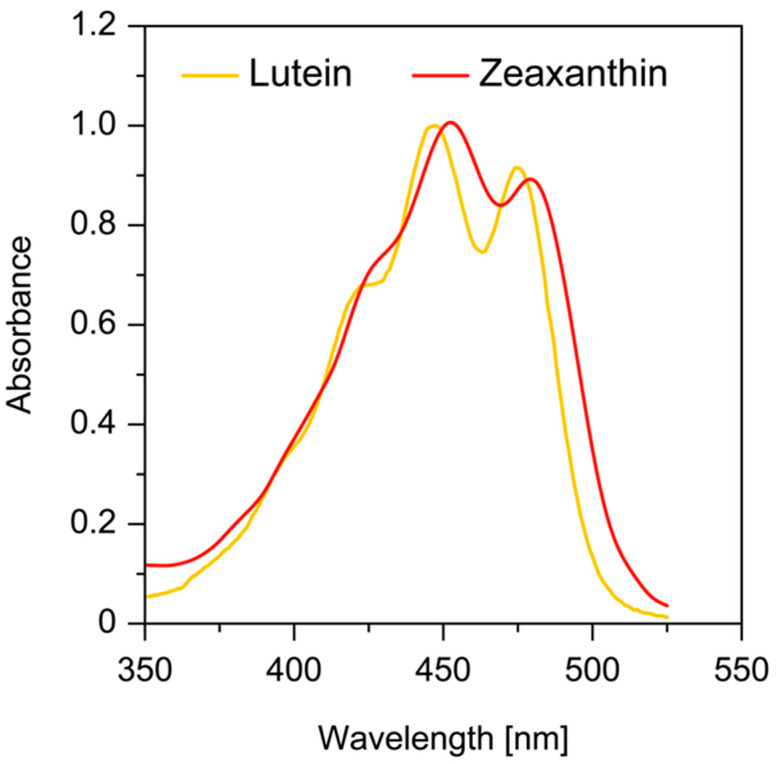
Absorption spectra of zeaxanthin and lutein in ethanol.

All ultraviolet (UV) light below 295 nm is absorbed by the cornea, and wavelengths between 300 and 315 nm and all UVA (315–400 nm) are absorbed by the crystalline lens [32]. Wavelengths between 400 and 700 nm (visible light) and 700 and 1400 nm (infrared radiation) are readily transmitted by the human ocular structures to the retina (Figure 1). Thus, almost all UV light is absorbed before it reaches the retina. However, the shortest wavelength of visible light (400–500 nm), which is transmitted by the lens, carries the highest energy per photon and is associated with photochemical damage to the retina. The human eye has developed two mechanisms that attenuate the transmission of blue light. One of them is the prereceptoral attenuation of blue light transmission by the lens, which becomes more yellow with age. The second is the additional protection obtained by a macular pigment filter mechanism. Macular xanthophylls help to absorb blue light, with peak absorption at approximately 450 nm (Figure 2). It has been shown that these macular xanthophylls may filter out approximately 40% of blue light [23,33]. The prereceptoral position of macular xanthophylls is perfect for protecting underlying photoreceptors.

For pigments in the same organic solvent, the absorption spectrum for zeaxanthin is similar to that of lutein and only shifts by approximately 5 nm in the long-wavelength region (Figure 2). Additionally, both xanthophylls, when dissolved in hydrated organic solvents, may form aggregated molecular structures, namely J- and H-type aggregates [34,35]. In H-type aggregates, carotenoid molecules are parallel to each other and are closely packed, whereas carotenoid molecules within J-type aggregates form head-to-tail arrangements with a looser organization. When incorporated into the lipid bilayers, xanthophylls appear mostly in monomeric form at concentrations below 0.5 mol% with respect to lipids. At higher concentrations, they can form aggregated structures (mostly H-type aggregates). The formation of such aggregates significantly changes the absorption properties of macular xanthophylls. The main absorption band peak for H-type aggregates is shifted by approximately 70 nm in the short-wavelength region. In contrast, the J-type aggregates are characterized by a red-shift of ~20 nm in the long-wavelength region. Thus, the two forms of aggregates possess different absorption spectra: blue-shifted (H-type aggregates) and red-shifted (J-type aggregates). In lipid membranes, under the same experimental conditions, the fraction of monomers observed for lutein was lower by 10–30% as compared with that for zeaxanthin [36]. Many factors, such as the solvent/water ratio, pH, temperature, the initial concentration of carotenoids, and the structure of carotenoids, have an influence on what type of aggregate is formed by carotenoids [34,37,38,39]. It is well known that the presence of two hydroxyl groups promotes the H-type organization of aggregates. H-type aggregates require a symmetrical carotenoid, and a card-pack arrangement is stabilized by a hydrogen-bonding network [40]. When the hydrogen-bond formation is prevented, e.g., by esterification or a lack of end-ring functional groups, J-type aggregates may be formed. Zeaxanthin, which is less polar than lutein, easily forms H-type aggregates in water/ethanol mixtures as compared with lutein [40]. The effects of the aggregation of lutein and zeaxanthin on their spectroscopic properties in the lipid environment in the context of blue light filtration have not been studied.

The role of carotenoids as a blue light filter is not fully understood. It is not clear whether macular xanthophylls are important for the reduction of chromatic aberration and light scattering (visual performance) or for the absorption of blue light before it excites potent photosensitizers present in the photoreceptor layer and retinal pigment epithelium (antioxidant performance). Many authors suggest that macular xanthophylls may be involved in the protection of the retina through many mechanisms, not just one function. The density of blue cones rises as the macular xanthophylls increase toward the center of the fovea. The reduction of light captured through blue light filtering in this region makes sense because the foveola is free of blue cones. It is well known that several carotenoids, including zeaxanthin and lutein, located in the oil droplets of birds’ eyes, work as high-energy light cut-off filters [41,42]. These carotenoid oil droplets are located within the inner segment of the retina. Colored oil droplets of bird’s eyes with appropriate carotenoid content probably provide protection against short-wavelength light and narrow the spectral bandwidth of the cones. A similar function may be performed by the zeaxanthin present in the central fovea, where only green and red cones exist. The foveal center of the human retina is insensitive to blue light. Müller cells (glial cells) in this central part of the fovea are thought to act as optical fibers [43,44] that transfer light without scattering to the photoreceptor layer and cut off blue light. Initially, macular xanthophylls were thought to be located in Henle’s fiber layer (foveal portion of the outer plexiform layer), but now it is widely accepted that Müller cells may be the sites of macular pigment deposition [45]. Macular telangiectasia (MacTel) type 2, which is a rare eye disease affecting the fovea, shows more evidence that Müller cells are the storage sites for macular xanthophylls. MacTel type 2 retinas demonstrate high zeaxanthin reduction in the central part of the fovea and this central depletion is correlated with the loss of Müller cells [46,47]. This suggests that Müller cells are crucial for the storage of zeaxanthin. In the foveal center, where zeaxanthin is located at high density, specialized glial Müller cells with yellow pigment form optical fibers with a blue light filter that concentrates green and red light onto cones [43]. It should be noted here that Müller cells span the entire retina, contacting all neuronal cell types, and contribute to the outer and inner limiting membranes and, therefore, serve structural, optical, and nutritional functions. Additionally, they might take part in the cone regeneration of cone all-trans-retinal [48]. Interestingly, Charbel Issa et al. [46] analyzed the macular density of patients with MacTel type 2 who had xanthophyll supplementation for several years and found a higher concentration of xanthophylls than average outside the central retina but not in the region of the most pronounced xanthophyll loss. The authors concluded that storage of retinal xanthophylls may be still possible but not in the areas affected by disease. This indicates that, in retinas affected by MacTel2, the transport and accumulation of macular xanthophylls, especially zeaxanthin, is disrupted in the central part of the macula.

### 2.2. Ability to Quench the Excited State of Oxygen (Singlet Oxygen)

Macular xanthophylls are very efficient at quenching singlet oxygen as compared with vitamin E, which is another lipid-soluble antioxidant present in the retina. It is common knowledge that a longer conjugated double bond system is associated with a greater singlet oxygen quenching rate constant in organic solvents [25,49]. Thus, lutein with 10 conjugated double bonds is 50% less efficient in the physical quenching of singlet oxygen in organic solvents than zeaxanthin with 11 conjugated double bonds [25] (see Table 1). The physical singlet oxygen quenching by carotenoids in the organic solvent depends on the triplet energy level of the carotenoid molecule and is very efficient for carotenoids with more than 11 conjugated double bonds. Physical singlet oxygen quenching takes place when the triplet energy level of a carotenoid is very near or below that of singlet oxygen. In this case, it is possible for energy to transfer from the singlet oxygen state to the carotenoid triplet excited state. Next, energy can be thermally dissipated through the non-radiative transition from the carotenoid triplet excited state to the carotenoid ground state. In this type of quenching, macular xanthophylls are not consumed and their structures remain intact.

Although the mechanism of singlet oxygen quenching by macular xanthophylls in simple organic solvents has been extensively studied, the same physical mechanism of the singlet oxygen quenching by both macular pigments in the lipid membrane systems has been investigated less extensively. Molecular organization of macular xanthophylls in lipid membranes and xanthophyll–lipid membrane interactions are crucial for proper antioxidant functioning. Singlet oxygen quenching may be efficient only when macular xanthophylls exist in monomeric form. For large aggregates, the ability to quench the excited state of oxygen is reduced to zero. The singlet oxygen quenching rate constants for macular xanthophylls in the lipid environment have been investigated by several authors (see Table 2). Cantrell et al. investigated the quenching of singlet oxygen by lutein, zeaxanthin, and other carotenoids in a model membrane system formed with dipalmitoylphosphatidylcholine (DPPC) [29]. Carotenoids were incorporated in the lipid phase of unilamellar liposomes, whereas the singlet oxygen was generated by two sensitizers: water-soluble sensitizer (rose bengal) and lipid-soluble sensitizer (4-(1-pyrene)butric acid). The second-order rate constant for the quenching of singlet oxygen by lutein incorporated within the DPPC membrane was a little lower than the constant observed for zeaxanthin (Table 2). Additionally, the authors indicated that singlet oxygen quenching decreases with an increasing zeaxanthin concentration [29]. The authors did not observe the same behavior for lutein and other studied carotenoids. Because the quenching of singlet oxygen by carotenoids in lipid bilayers is more complex than in organic solution, several factors affecting this process must be considered, including orientation in the lipid bilayer and the organization of carotenoid molecules. Meso-zeaxanthin, a stereo isomer of zeaxanthin, with 11 conjugated double bonds, should possess the same antioxidant properties as zeaxanthin. However, meso-zeaxanthin was found to be a better antioxidant in the presence of GSTP1 than zeaxanthin [50] and has the strongest ability to quench singlet oxygen in organic solution [51]. Indeed, it has been shown that a mixture of the three carotenoids (meso-zeaxanthin, zeaxanthin, lutein) has a greater singlet oxygen quenching ability than any of the individual macular xanthophylls [51].

### 2.3. Ability to Quench the Excited Triplet State of Photosensitizers

**Figure 3:**
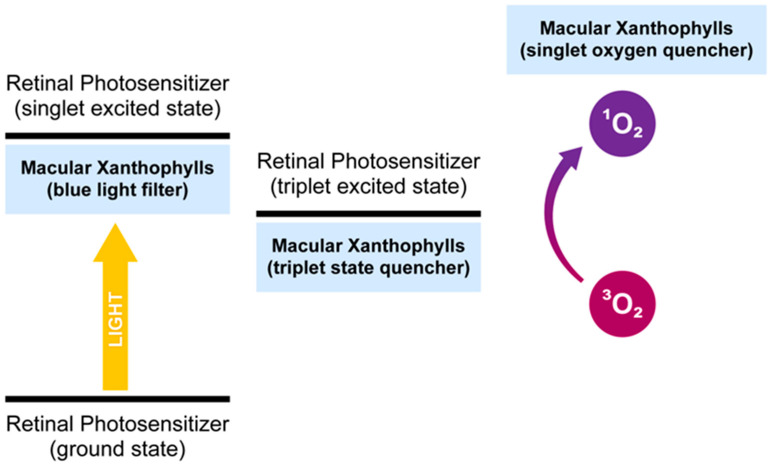
Triple protection of macular xanthophylls against singlet oxygen.

The macular xanthophylls accept deactivation of already existing singlet oxygen but may also prevent the generation of this excited form of oxygen. In fact, macular xanthophylls offer triple protection to cells against single oxygen: (i) they quench singlet oxygen directly, (ii) they prevent the generation of singlet oxygen by quenching the excited triplet state of photosensitizers, and (iii) they prevent the photosensitizers from being excited (blue light filtration function mentioned in Section 2.1) (Figure 3). The ocular chromophore may be in a long-lived excited triplet state. The energy from this long-lived state may be transferred to the ground state of oxygen (triplet oxygen), creating an excited site of oxygen (singlet oxygen). Carotenoids may transfer energy from the excited triplet state of photosensitizers to their own excited triplet states and then lose energy in the form of heat. The energy transfer only occurs over the short distance between the photosensitizer and the quencher. This process is well known for carotenoids in thylakoid membranes [52,53,54] but not for the retina. In the retina, singlet oxygen can be generated by all-trans-retinal phosphatidylethanolamine adduct (A2E) and other bis-retinoid constituents in the retinal pigment epithelia (RPE) [55,56]. Lutein and zeaxanthin are present in human RPE, but at a lower quantity than in prereceptoral layers. The RPE controls xanthophyll transport. Additionally, meso-zeaxanthin production from dietary lutein by RPE65 enzymes occurs in the RPE. The quenching of the triplet state of chlorophyll by carotenoids is crucial for photoprotection against singlet oxygen in photosynthetic organisms [53,54]. Unlike in photosynthetic organisms, photosensitizer–xanthophyll triplet-triplet energy transfer is not known to occur in the retina. Free all-trans-retinal is another potent photosensitizer photoactivated by UVA and blue light. The quantum yield of the triplet state of all-trans-retinal is high [57]. All-trans retinal is released in photoreceptor outer segments from the photoactivated visual pigment and converted back to 11-cis retinal within RPE as part of the regeneration process. Problems occur when all-trans-retinal is not efficiently cleared from retinal outer segment discs. Several studies indicate that all-trans-retinal, which is the precursor of A2E, has higher phototoxicity than A2E. Thus, all-trans-retinal bound to phospholipids has less photosensitizing activity than free all-trans-retinal, and the production of A2E may be a protective mechanism in the retina [58,59].

## 3. Chemical Antioxidant Actions of Macular Xanthophylls

### 3.1. Chemical Reactions with Singlet Oxygen

The chemical quenching of singlet oxygen by carotenoids leads to oxidation of the quencher. This oxidation destroys the intact structure of carotenoids and requires de novo synthesis or recycling of the pigment molecule. Although the chemical quenching constitutes a minor part of singlet oxygen quenching by carotenoids [60], several authors have reported oxidation products for xanthophylls in human tissue [61,62,63,64]. Endoperoxides and different aldehydes were identified as the oxidation products of lutein and zeaxanthin. Ramel et al. demonstrated that zeaxanthin endoperoxide accumulated at a slower rate than the lutein oxidation product in the leaves of plants exposed to photooxidative stress. Zeaxanthin endoperoxide occurred after 30 min, whereas the lutein endoperoxide occurred during the first 15 min after illumination [65]. Ramel et al. proposed [65] that zeaxanthin oxidizes less rapidly by singlet oxygen than lutein and β-carotene. Similarly, carotenoid consumption in the lipid bilayer under the influence of light and oxygen and in the presence of toluidine blue as a photosensitizer was higher for β-carotene than for zeaxanthin [54]. Other studies also indicate that carotenes usually decompose more rapidly than xanthophylls [66,67]. Consistent with this, it was found that both zeaxanthin and lutein attenuate the photooxidation of A2PE without any evidence of oxidation during this process, and they would both participate in physical quenching with minimal turnover. For chloroplasts of green leaves, faster β-carotene degradation is associated with rapid turnover of these pigments. Unlike β-carotene, slow turnover was found for xanthophylls [68]. All of these results may imply that the degradation and turnover of carotenes (nonpolar carotenoids) are greater compared with xanthophylls (dipolar carotenoids) and that zeaxanthin is less rapidly oxidized by singlet oxygen than lutein [65].

### 3.2. Direct Reactions with Other ROS

Macular xanthophylls not only quench singlet oxygen but also act as scavengers of the superoxide anion radical (O_2_^•−^) and hydroxyl radical (OH^•^) [68,69,70,71]. The superoxide anion radical and hydroxyl radical are oxidants that cause lipid peroxidation, resulting in damage to the integrity of biological membranes. Carotenoids may accept or give the electron from the polyene chain; thus, oxyradicals cannot induce the oxidation of lipid and other molecules in cells. Macular xanthophylls react with superoxide via electron transfer to generate the radical cation or anion:

MX + O_2_^•−^ → MX^•−^ + O_2_ (1)

MX + O_2_^•−^ (+2H^+^) → MX^•^ + H_2_O_2_ (2)

The mechanism against the hydroxyl radical mostly occurs through formation of the adduct radical, as shown in the equation below:

MX + OH^•^ → (MX − OH) ^•^ (3)

Trevithick et al. have used electron spin resonance–spin trapping to evaluate the scavenging ability of different carotenoids, including macular xanthophylls [70]. They reported that both lutein and zeaxanthin are better hydroxyl radical scavengers than superoxide anion radical scavengers and that zeaxanthin scavenged the hydroxyl radical more effectively than lutein. The same result was obtained by Rodrigues et al. [72], who showed that, in liposomes, the hydroxyl radical scavenging capacity of lutein is lower as compared with zeaxanthin. Conversely, Boehm and coauthors came to a different conclusion, showing that, in cellular studies, lutein is a better protector against hydroxyl radicals generated by gamma radiation [71].

### 3.3. The Chain-Breaking Activity

Macular xanthophylls protect the retina from photochemical damage by preventing lipid peroxidation (quenching singlet oxygen and trapping free peroxyl radicals). The retina is vulnerable to oxidative damage due to (i) exposure to light, (ii) a high concentration of oxygen, (iii) an abundance of photosensitizers, and (iv) a high concentration of polyunsaturated fatty acids (PUFAs) [73,74]. The high concentration of PUFAs is found in rod outer segments, where docosahexaenoic acid (DHA, 22:6n-3) reaches 50% of the total phospholipids [75]. Additionally, very long-chain PUFAs (VLC-PUFAs) synthesized from PUFA precursors (22:4n-6 and 22:5n-3 phospholipids) in the retina are also present [76,77,78]. Carotenoids might react with alkoxy radicals, lipid peroxy radicals, and alkyl radicals derived from polyunsaturated fatty acid oxidation and inhibit the formation of hydroperoxides. Both xanthophylls—lutein and zeaxanthin—show similar capacities to scavenge peroxy radicals [72]. However, the chain-breaking activity of macular xanthophylls is greater than that of the retinal antioxidant, vitamin E.

## 4. Xanthophyll–Membrane Interactions

### 4.1. Transmembrane Location

**Figure 4:**
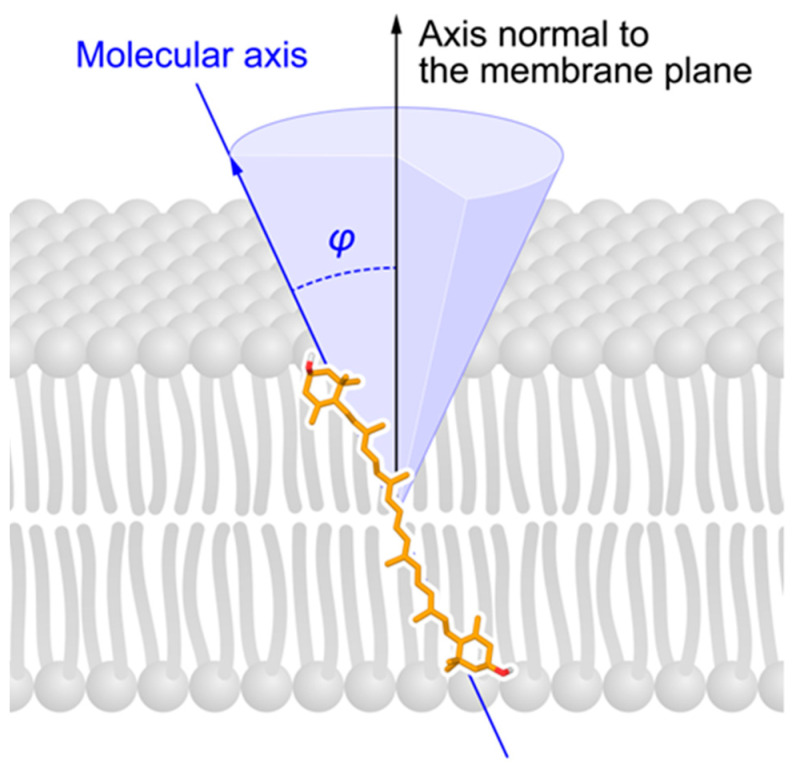
Schematic drawing of the transmembrane orientation of zeaxanthin and lutein in the lipid bilayer membrane (see [79] for more details).

The first approach to determine the orientation of the macular xanthophyll molecule within the lipid membrane was based on the measurements of the linear dichroism of carotenoids embedded in the oriented lipid multi-bilayers [36]. In these measurements, a system of over 100 bilayers of lipids was placed on the glass substrate, and the UV–visible absorption spectra of polarized light parallel and perpendicular to the incidence plane of the lipid samples containing carotenoids were analyzed. This model system comprising stacked lipid bilayers seems ill-suited for the calculation of the precise orientation of macular xanthophylls in the lipid bilayer because analyzed molecules could localize in the intermembrane spaces. In the present studies, Gruszecki’s group analyzed a single lipid bilayer membrane, which meant that the fraction of molecules did not localize in the space between lipid bilayers [79]. The results of the molecular spectroscopy analyses (including linear dichroism and photoselection experiments based on light absorption, fluorescence, and resonance Raman scattering, combined with the computational molecular dynamics studies) show that macular xanthophylls—lutein and zeaxanthin—span the lipid bilayer and that two hydroxy groups are located in the opposite polar zones of the membrane [79]. The values of the average angle between the transition dipole moment and the axis normal to the plane of the membrane prepared from dimyristoylglycerophosphatidylcholine (DMPC) were very similar (i.e., 41.8° for zeaxanthin and 42.7° for lutein) [79]. It should be noted that an electronic transition dipole moment of carotenoids is not exactly oriented along the major axis of the polyene chain. There is approximately 10° ÷ 15°  between the dipole transition and the molecular axis defined by the direction of the conjugated double bond system [80]. Thus, both zeaxanthin and lutein adopt transmembrane orientation with respect to the plane of the membrane (Figure 4). Additionally, electron paramagnetic resonance spin-labeling studies have shown similar effects of lutein and zeaxanthin on the physical properties of the lipid bilayer, supporting the transmembrane orientation of these two macular pigments [81,82,83]. The same transmembrane orientation of zeaxanthin and lutein in the model lipid membrane cannot explain the different spatial distributions and different functions of these pigments in the retina. This means that to explain the differences in the spatial distribution of lutein and zeaxanthin in the macula (a large amount of zeaxanthin in the central fovea and the presence of only lutein in the peripheral retina) [62], different molecular interactions or mechanisms functioning at the molecular level have to be considered. Structurally, zeaxanthin and lutein are similar. Both are dihydroxy xanthophylls with small differences in their ring structure. Zeaxanthin has two β-rings while lutein has one β-ring and one ε-ring. Due to the different location of a double bond in the ε-ring, lutein has a shorter conjugated double bond system than zeaxanthin. It is well known that most of the carotenoids common in plants and algae have two β-rings. Carotenoids with one β-ring and one ε-ring are also commonly present in plants and algae, but pigments with two ε-rings are rare [84]. All forms of vitamin A have a β-ionone ring. Canthaxanthin, which is used as an oral tanning agent and accumulates at a high dose in the foveal and perifoveal region in the form of crystals, is also a β-ionone ring carotenoid [85]. Recently, molecular dynamic simulation studies on the intercalation of lutein into the lipid bilayer have shown that lutein intercalates into the bilayer preferentially with its β-ring [86]. Conversely, the ε-ring docks in the active site of RPE65, the enzyme responsible for the conversion of lutein to meso-zeaxanthin in the retina [87], and docks in the cavity of the lutein-specific steroidogenic acute regulatory domain protein (StARD3) [88].

### 4.2. Location in the Most Vulnerable Regions of Lipid Membranes

**Figure 5:**
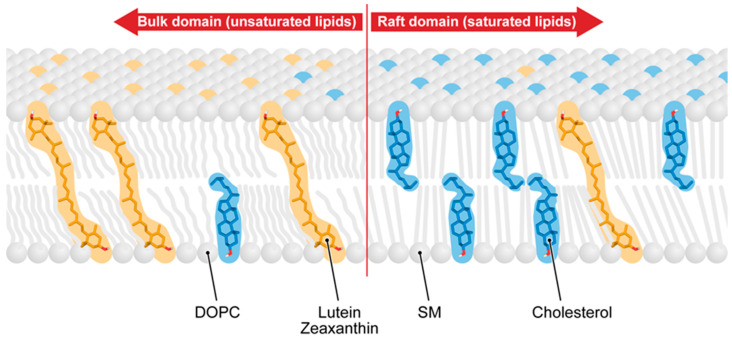
Schematic drawing showing the distribution of macular xanthophylls between the unsaturated bulk domain and the saturated raft domain in lipid-bilayer membranes. (see [90,95] for additional details). DOPC, dioleoylphosphatidylcholine; SM-sphingomyelin.

The macular xanthophyll content seems to be high in the human eye retina, but its concentration in the lipid bilayer portion of the membrane is much lower than 1 mol% [89]. Therefore, to effectively act as an antioxidant, macular xanthophyll should be selectively located in close proximity to the protected vulnerable molecules. It was shown that the lateral membrane organization into membrane domains ensures colocalization of macular xanthophylls with protected polyunsaturated phospholipids and rhodopsin. In model membranes formed from an equimolar ternary mixture of dioleoylphosphatidylcholine(DOPC)/sphingomyelin(SM)/cholesterol, called a raft-forming mixture, containing raft domains (detergent-resistant membranes (DRMs)) and surrounding bulk domains (detergent-soluble membranes (DSMs)), lutein and zeaxanthin were around eight times more concentrated in the unsaturated bulk domain (isolated as DSM) and excluded from the raft domain (enriched in saturated lipids and cholesterol and isolated as DRM) [90] (Figure 5). The lipid composition of photoreceptor outer segment (POS) discs is similar to that of the raft-forming mixture, with a nearly equimolar concentration of unsaturated fatty acids (DHA), saturated fatty acids (myristoyl + palmitoyl + stearoyl), and cholesterol [91], and raft domains were isolated as DRMs from POS discs [91,92,93,94]. We also investigated the distribution of macular xanthophylls between domains in the POS membrane model, which consisted of an equimolar ternary mixture of 1-palmitoyl-2-docosahexaenoylphosphatidylcholine/distearoylphosphatidylcholine/cholesterol [95]. Lutein and zeaxanthin were around fourteen times more concentrated in polyunsaturated DHA enriched and isolated as a DSM fraction and excluded from the raft domain. Monopolar β-cryptoxanthin showed a distribution similar to that of macular xanthophylls, while nonpolar β-carotene was more uniformly distributed between domains (see [96] for additional discussion).

Human retina membranes contain high amounts of polyunsaturated fatty acids such as DHA, PUFAs, and VLC-PUFAs, which, together with rhodopsin, are isolated from POS disc membranes as a DSM fraction [73,92,94,97,98]. Colocalization of rhodopsin with polyunsaturated phospholipids is functionally justified because rhodopsin requires these lipids for its activity [99,100,101]. DRMs and DSMs isolated from cell membranes have similar lipid compositions as raft and bulk domains, respectively, and are related to these membrane domains [102,103]. All these data indicate that in the membranes of POS discs, lutein and zeaxanthin should be located and concentrated in the bulk domain, where polyunsaturated lipids and rhodopsin are also located. Polyunsaturated lipids located in the bulk domain of POS disc membranes are extremely susceptible to lipid peroxidation. Additionally, photoactivation of rhodopsin leads to the formation of all-trans-retinal (see Section 2.3), which can act as a photosensitizer. Although the co-localization of rhodopsin with polyunsaturated phospholipids has a functional purpose, it creates a dangerous situation for both, especially during illumination, when ROS can be produced by photosensitizers. During biological evolution, macular xanthophylls were chosen as effective protectors of the retina because they can neutralize photosensitizers and ROS. Localization of macular xanthophylls in domains rich in unsaturated lipids is ideal if they are to act as a lipid antioxidant, which is the most accepted mechanism through which lutein and zeaxanthin protect the retina from age-related macular diseases [104,105,106]. This is possible because of the domain structure of the POS membrane and the ability of these domains to select and exclude specific classes of lipids and proteins. The distribution of lutein and zeaxanthin between bulk and raft domains is the same and cannot differentiate the antioxidant potency of these two macular xanthophylls.

### 4.3. Membrane Solubility

Both macular xanthophylls have poor aqueous solubility but high solubility in lipid membranes, much higher than nonpolar carotenoids. The incorporation of zeaxanthin and lutein into phospholipid liposomes (the multilamellar vesicles) was studied by Socaciu et al. [107]. The multilamellar vesicles were prepared from DPPC and a mixture of phospholipids DPPC:DPPE(1,2-dipalmitoyl phosphatidyletanolamine):DPPS(1,2-dipalmitoyl diphosphatidylserine). The authors observed greater incorporation of macular xanthophylls than nonpolar β-carotene. Additionally, the incorporation of all studied carotenoids (zeaxanthin, lutein, β-carotene) was impeded by membranes loaded with cholesterol [107]. The incorporation of zeaxanthin, lutein, canthaxanthin, and β-carotene separately into membranes of small unilamellar vesicles made from the EYPC (egg yolk phosphatidylcholine) [88] was the highest for lutein. Incorporation of zeaxanthin was weaker than that for lutein but much higher than for β-carotene and canthaxanthin. Similarly, the higher incorporation efficiency of macular xanthophylls than that of β-carotene and canthaxanthin was observed for pig liver microsomes and for human RPE cultured cells [108]. Moreover, the accumulation of zeaxanthin into cell membranes was higher than that for lutein [108,109]. The greater incorporation yield of macular xanthophylls into liposomes and RPE cells than β-carotene suggests high stabilization of xanthophyll molecules in the membranes of the human retina due to their transmembrane orientation, enhanced by strong anchoring of hydroxyl groups at opposite membrane surfaces. Such a location can explain their very slow removal from the human retina (up to six months) after discontinuation of their supplementation [110,111]. For comparison, the rat retina is capable of completely replacing its cholesterol, which is anchored at the membrane surface by only one hydroxyl group, every week, and, in humans, this replacement may be even more rapid [112]. We demonstrated that at high concentrations, the effects of zeaxanthin and lutein on membrane structure and dynamics are practically the same. Both lower the temperature and broaden the main phase transition of the phospholipid membrane [83,113,114], decrease the oxygen diffusion concentration product in the hydrocarbon region of the lipid bilayer [81,83,115,116], and increase the hydrophobicity in the central region of the lipid bilayer [117]. Finally, both macular xanthophylls increase the order and decrease the alkyl chain motion in fluid phase membranes [83,115,116]. We can conclude that zeaxanthin and lutein are similar in their function as modifiers of lipid bilayer membranes.

## 5. Mechanisms Maintaining the Intact Structure of Macular Xanthophylls

As mentioned in Section 4.3, Landrum et al. [110] reported that after discontinuation of lutein supplementation, the lutein concentration in the serum decreased relatively quickly, whereas the level of lutein in the retina remained the same for a long time. These results suggest that macular xanthophylls are highly stable in retina membranes. In the retina (an environment that is constantly exposed to light, has a high oxygen concentration, contains photosensitizers, and is rich in polyunsaturated phospholipids), mechanisms maintaining the intact structure of macular xanthophylls should exist. It was demonstrated by several authors that carotenoids, vitamin E, and vitamin C are synergistic antioxidants [118,119,120,121,122,123,124,125]. Vitamin E is known as the most effective free radical scavenger, better than macular xanthophylls. The combination of zeaxanthin and vitamin E exerts strong synergistic protection against singlet oxygen-induced lipid peroxidation. This synergistic effect may be explained in terms of protection of the intact zeaxanthin structure by effective scavenging of free radicals by vitamin E, therefore allowing zeaxanthin to quench the primary oxidant, singlet oxygen, effectively by the physical protective mechanism [124,126,127]. Additionally, the oxidized forms of zeaxanthin can be re-reduced by water-soluble vitamin C [125]. Interestingly, vitamin E displays a radial distribution in the retina similar to that of zeaxanthin [24,128]. The effects of different carotenoids, vitamin E, and their combination on lipid peroxidation induced by a lipid-soluble peroxyl radical generator (AMVN) were studied by Stahl et al. [129]. Separately, without vitamin E, the inhibitory effect of carotenoids decreased in the following order: lycopene > α-carotene > β-cryptoxanthin > zeaxanthin = β-carotene > lutein. The authors demonstrated that mixtures of carotenoids with different combinations were more effective than single compounds. It can be concluded that many factors, including the site and the rate of radical production, localization of carotenoid molecules, their concentration, and unsaturation of the lipid membrane, significantly impact the results obtained by different researchers in studies of the protection of membrane oxidation provided by carotenoids and their synergistic action with other antioxidants.

## 6. Mechanisms of Transport and Delivery to the Retina

Numerous studies have provided evidence of a correlation between increased dietary macular xanthophyll intake and the increase in macular pigment density [110,111,130]. Generally, in serum, nonpolar carotenoids are transported mostly in low-density lipoproteins (LDLs), whereas high-density lipoproteins (HDLs) are specific transporters of lutein and zeaxanthin into the retina. Thomas and Harrison demonstrated in in vitro studies that zeaxanthin is delivered to the human RPE cells (ARPE-19) mostly by HDL, whereas lutein via LDL [131]. Thus, zeaxanthin is taken up from HDL via a scavenger receptor B1 and lutein is preferentially delivered by LDL and taken up via an LDL receptor [132]. It was proposed that both dietary xanthophylls (lutein and zeaxanthin) enter the macula through the RPE layer via the choroidal vasculature [62]. However, another supplying mechanism is also possible through the retinal vasculature if xanthophylls are accumulated in the Müller cells. The third macular xanthophyll, meso-zeaxanthin, which is not present in most diets, is synthetically made from lutein by the retinal pigment epithelium-specific 65 kDa protein (RPE65) enzyme in the RPE [87]. The retinal xanthophyll transporters (xanthophyll-binding proteins) are also different for two macular xanthophylls and have been identified and characterized by Bernstein and co-workers. The lutein-specific StARD3 protein binds lutein [88,133,134,135], whereas the zeaxanthin-specific glutathione S-transferase (GSTP1) binds zeaxanthin [134,136]. These proteins are selective transporters responsible for the specific and different distribution of lutein and zeaxanthin in the retina. GSTP1 has been shown to be localized within the outer plexiform layer (Henle’s fiber layer) and inner plexiform layer [133], while StARD3 is primarily located within cone photoreceptor inner segments and neurons [135]. Only GSTP1 distribution correlates well with the predominant localization of macular xanthophylls [15,137]. It is also noteworthy here that antibodies to StARD3 do not colocalize with the immunohistochemical markers of Müller cells, glutamine synthetases [135], so Müller cells probably are not the lutein storage site. The protective effect of GSTP1 with zeaxanthin, incorporated into model membranes formed from EYPC, on lipid peroxidation was studied by Bhosale and Bernstein; membranes were subjected to oxidative stress induced by water-soluble 2,2′-azobis(2-amidinopropane) hydrochloride (AAPH) and lipid-soluble AMVN lipid peroxyl radical generators [50]. The authors found a synergistic antioxidant effect of the action of zeaxanthin and meso-zeaxanthin in combination with GSTP1 against both lipid peroxyl radical generators (AMVN and AAPH).

## 7. Concluding Remarks

The first separation of a carotenoid from a human retina was performed by Bone et al. in 1985 [138]. The authors identified that the yellow pigments of the macula were composed of two dietary carotenoids: zeaxanthin and lutein. In 1993, Bone et al. [139] found that retinal zeaxanthin is a mixture of two stereoisomers, zeaxanthin and meso-zeaxanthin. Unexpectedly, the fovea contains around twice as much zeaxanthin and meso-zeaxanthin as lutein, but this ratio is reversed in the periphery of the human retina [139]. Recently, Li et al. found that the meso-zeaxanthin and zeaxanthin-to-lutein ratio may be even greater than 2:1 and reaches values of around 9:1 in the center of the fovea and 4:1 at a foveal location 200 μm in diameter [17] (see Table 3). Thus, the fovea favors zeaxanthin, but the mechanism of this special and selective accumulation is unknown. Similarly, the reason that this happens is unknown. The remarkable special distribution of macular xanthophylls (the ratio of zeaxanthin-to-lutein declines with the increasing eccentricity) and conversion of lutein to meso-zeaxanthin in the retina indicate that zeaxanthin is more important for the retina. In previous publications, we directed our attention toward the differences between polar carotenoids (xanthophylls) and nonpolar carotenoids (carotenes) [83,116,140,141]. We can conclude that xanthophylls and not carotenes are selectively present in the retina for the following reasons: (i) high membrane solubility, (ii) transmembrane orientation, (iii) high chemical stability, and (iv) its location in the most vulnerable regions of the lipid membrane. All of these properties distinguish zeaxanthin and lutein from other dietary carotenoids. During evolution, lutein was chosen as a compromise (i.e., trade-off) molecule for the retina. The human diet contains mainly lutein, and dietary intake of zeaxanthin is much lower than that of lutein (the zeaxanthin-to-lutein molar ratio varies from 1:12 to 1:5). Thus, during evolution, the mechanisms developed to produce zeaxanthin from lutein as a meso-zeaxanthin.

It is also important to understand why zeaxanthin is located in the central part of the retina, which is the region where the risk of oxidative damage is the highest. The studies involving lipid membranes explain how the lipid bilayer affects the organization of macular xanthophylls, including orientation (transmembrane versus parallel), localization (distribution between membrane domains), solubility (monomers versus aggregates), and even optical properties (monomers versus blue-shifted aggregates and red-shifted aggregates). The orientations of lutein and zeaxanthin molecules in the lipid bilayer are similar (transmembrane, tilted approximately 40° to the normal) [79]. Such transmembrane orientations strongly influence the local diffusion concentration product of oxygen [81,115,116] and penetration of water into the lipid bilayer [116,117], as well as membrane fluidity [82,142]. The distribution between different lipid membrane domains is roughly the same for lutein and zeaxanthin (they are 8 ÷ 10 times more concentrated in the unsaturated bulk domain than in the saturated raft domain) [96,116]. Both macular xanthophylls are easily soluble in pure phospholipid model membranes and are poorly soluble in lipid bilayers with high cholesterol content [90,95,96,107]. However, the accumulation of zeaxanthin into cell membranes was shown to be higher than that for lutein [108,109]. Finally, based on the presented materials, we can state that, in terms of its antioxidant properties, zeaxanthin is a better lipid-soluble singlet-oxygen quencher and is less rapidly oxidized by singlet oxygen than lutein [25,26,27,29,30,31,65].

Of the many carotenoid studies, only a few of them compare the antioxidant properties of zeaxanthin with those of lutein and compare their different behaviors in the human retina. Such studies may help to explain the unique accumulation of zeaxanthin in the fovea (see reference [140] for review). Studies of carotenoid–membrane interactions (including our own studies [79,83,95,116,117]) focused on differences between nonpolar (carotenes) and polar carotenoids (zeaxanthin and lutein), possibly because no significant differences were found in the interactions with membranes between zeaxanthin and lutein. A recent paper from Bernstein’s laboratory sheds light on the crucial need for an explanation as to why “zeaxanthin may play a more important role than lutein in human macular health and disease” [17].

## Figures and Tables

**Table 1:** Second-order singlet oxygen quenching rate constant of macular xanthophylls in organic solvent.

Sensitizer	Organic Solvent	Zeaxanthin	Lutein	Ref.
Endoperoxide of 1,4-dimethylnaphthalene	Deuterochloroform, CDCl_3_	1.9 × 10^9^ M^−1^ s^−1^	0.8 × 10^9^ M^−1^ s^−1^	[26]
unspecified	Benzene	12 × 10^9^ M^−1^ s^−1^	6.64 × 10^9^ M^−1^ s^−1^	[25]
3-(1,4-epidioxy-4-methyl-1,4-dihydro-1-naphthyl	Ethanol/chloroform/D_2_O (50:50:1, *v*/*v*/*v*)	11.2 × 10^9^ M^−1^ s−^1^	8.1 × 10^9^ M^−1^ s^−1^	[27]
*Meso*-tetraphenylporphyrin	Benzene	12 × 10^9^ M^−1^ s^−1^	11 × 10^9^ M^−1^ s^−1^	[28]

**Table 2:** Second-order singlet oxygen quenching rate constant of macular xanthophylls in lipid environment.

Sensitizer	Model Membrane System	Zeaxanthin	Lutein	Ref.
Rose bengal	unilamellar DPPC liposomes	2.3 × 10^8^ M^−1^ s^−1^	1.1 × 10^8^ M^−1^ s^−1^	[29]
4-(1-pyrene)butric acid	unilamellar DPPC liposomes	1.7 × 10^8^ M^−1^ s^−1^	0.82 × 10^8^ M^−1^ s^−1^	[29]

**Table 3:** The comparison between zeaxanthin and lutein.

	Zeaxanthin	Lutein
Ratio Diet Serum Foveola ^1^ Fovea ^1^ Periphery ^1^	Zea/Lut 1:12 ÷ 1:5 1:4 9:1 4:1 1:2	Lut/Zea 12:1 ÷ 5:1 4:1 1:9 1:4 2:1
End rings	two β-rings	one β-ring and one ε-ring
Peak absorption ^2^	451 nm	445 nm
Primary function	cone protection	rod protection
Binding protein	GSTP1	StARD3
Transport in serum	mostly in HDL	mostly in HDL
Singlet oxygen quenching rate ^3^	high	lower compared with Zea

^1^ Ref. [17]. ^2^ Absorption spectra of xanthophylls in ethanol. ^3^ See Table 1 and Table 2.
